# Development and Validation of the Adolescent Bystander Intervention Barrier Perception Scale in School Bullying

**DOI:** 10.3390/bs16010055

**Published:** 2025-12-29

**Authors:** Zheng Mao, Yisheng Yang

**Affiliations:** 1 School of Psychology, Inner Mongolia Normal University, Hohhot 010022, China; psy_mz@126.com; 2 Inner Mongolia Student Bullying Prevention Research Center, Tongliao 028000, China

**Keywords:** school bullying, bystander intervention, barrier perception, adolescents, scale development, measurement invariance

## Abstract

Based on the theoretical framework of psychological barriers among third-party bystanders in school bullying contexts, grounded in Protection Motivation Theory and Ecological Systems Theory, this study developed and validated the “Adolescent Bystander Intervention Barrier Perception Scale” (ABIBPS). The initial item pool was developed through literature review and semi-structured interviews, followed by item analysis, exploratory and confirmatory factor analyses across three samples (middle school students, *N* = 388; middle school students, *N* = 474; upper elementary school students, *N* = 547). Results revealed a robust two-factor structure comprising “Personal Risk and Fear Perception” and “Intervention Efficacy and Outcome Uncertainty.” The scale demonstrated measurement invariance across different age groups, good internal consistency reliability, structural validity, and criterion-related validity. Correlation analyses indicated that adolescent bystander intervention barrier perceptions were significantly negatively associated with prosocial behavior, positive youth development, intentional self-regulation, and self-esteem. This study provides a valid measurement tool for understanding the psychological barrier mechanisms of bystander behavior in school bullying, offering significant theoretical and practical implications for promoting active intervention behaviors among adolescents.

## 1. Introduction

School bullying, as a group phenomenon with profound negative impacts on adolescents’ physical and mental health, affects not only victims and perpetrators but also shapes the entire school social-ecological environment (Olweus, 1994; Salmivalli, 2010). In the process of school bullying, roles related to bullying behavior are termed participant roles in bullying, including bullies, victims, and bystanders (Salmivalli et al., 1996). Bystanders, as the “silent majority,” play a crucial yet often overlooked role (Salmivalli et al., 1996; Thornberg et al., 2012). Bystanders refer to individuals who are neither bullies nor victims but witness bullying incidents. According to Salmivalli et al.’s (1996) participant role theory, bystanders can be further categorized into: (1) followers, who assist bullies but do not initiate bullying; (2) reinforcers, who provide positive feedback to bullying behaviors through laughter, applause, etc.; (3) outsiders, who choose not to intervene and remain silent; and (4) defenders, who actively intervene in bullying through direct intervention, comforting victims, or seeking adult help. This classification breaks the traditional “bully victim” binary model, revealing the diversity of roles in bullying incidents (Salmivalli, 2010).

Research indicates that bystanders are present in approximately 85% of bullying incidents (Hawkins et al., 2001), and their responses significantly influence the persistence and severity of bullying behaviors. When bystanders choose to intervene actively, approximately 57% of bullying behaviors stop within 10 s (Hawkins et al., 2001); conversely, bystander support or indifference reinforces bullying behaviors, making them “normative behaviors” in school settings (Salmivalli et al., 2011; Sandstrom et al., 2013).

Despite the effectiveness of active intervention in reducing bullying, the proportion of bystanders who choose to intervene remains low in reality, with only 10–20% of bystanders taking intervention actions (Obermann, 2011; Pozzoli & Gini, 2010). This paradox has prompted exploration into bystander psychological mechanisms: what prevents adolescents from taking active intervention actions?

This study primarily builds the conceptual foundation of bystander intervention barriers on two core theoretical frameworks: Protection Motivation Theory and Ecological Systems Theory. Protection Motivation Theory (Rogers, 1975) posits that behavioral decisions in the face of threats are determined by two processes: threat appraisal (including severity and vulnerability) and coping appraisal (including response efficacy, self-efficacy, and response costs). In bystander contexts, intervention barriers primarily manifest as overestimation of intervention costs and underestimation of self-efficacy (DeSmet et al., 2016). Ecological Systems Theory (Bronfenbrenner, 1979) emphasizes that behavior is influenced by interactions across multiple system levels, directing our attention to multi-level factors influencing intervention decisions, including individual factors, peer factors, school factors, and broader socio-cultural factors.

**Figure 1:**
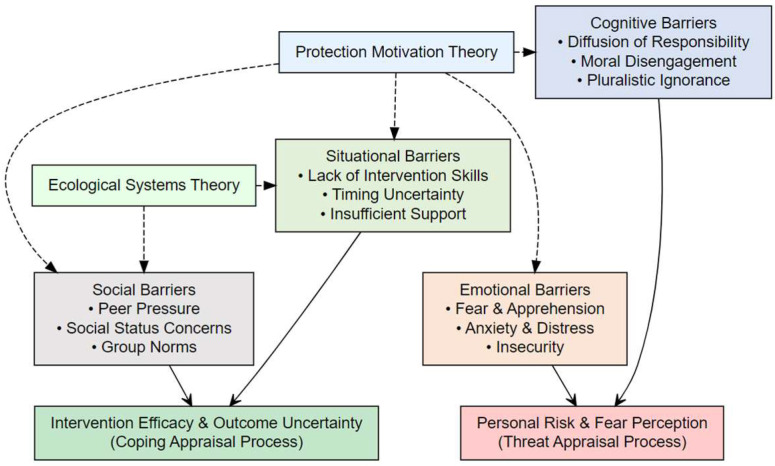
Classification Framework of Bystander Intervention Barriers.

Existing research has identified various factors affecting bystander intervention, which can be broadly categorized into four types (see Figure 1):

Cognitive barriers include diffusion of responsibility (Darley & Latané, 1968), moral disengagement (Thornberg & Jungert, 2013), and pluralistic ignorance (Sandstrom et al., 2013). When the number of bystanders increases, individuals’ perceived responsibility for intervention significantly decreases (Espelage et al., 2012); adolescents with higher levels of moral disengagement are less likely to take intervention actions (Thornberg & Jungert, 2013).

Emotional barriers involve fear, anxiety, and insecurity. Concerns about personal safety are a primary emotional factor hindering intervention (Thornberg et al., 2012), with adolescents worrying that intervention might make them the next victim (Doramajian & Bukowski, 2015).

Social barriers refer to group norms, peer pressure, and social status considerations. In classrooms where bullying behavior is viewed as “normal,” bystander intervention rates decrease significantly (Pozzoli et al., 2012); fear of losing social status or experiencing peer rejection is one of the main reasons adolescents choose not to intervene (Juvonen & Galván, 2008).

Situational barriers include lack of intervention skills, timing uncertainty, and insufficient environmental support. Even adolescents with intervention intentions often choose inaction due to not knowing how to intervene effectively (Palmer et al., 2015); school climate and teacher attitudes significantly influence bystanders’ intervention decisions (Jenkins & Nickerson, 2019).

Although these studies provide important insights, most focus on single or limited barrier factors, lacking systematic integration and measurement (Jenkins & Nickerson, 2019). Additionally, existing research is primarily based on Western samples, with limited understanding of adolescent bystander psychological barriers in Chinese cultural contexts.

Among existing measurement tools, such as the “Cyberbullying Defending Self-Efficacy Scale” (Bussey et al., 2020) and the “Bystander Intervention Scale” (Nickerson et al., 2014), the focus is mainly on positive factors promoting intervention, with less systematic examination of psychological barriers hindering intervention. According to behavior change theory, identifying and eliminating behavioral barriers is often more effective than merely enhancing promoting factors (Michie et al., 2011; Schwarzer, 2008). In the Chinese cultural context, there is still a lack of measurement tools specifically targeting adolescent bystander intervention barriers.

“Adolescent bystander intervention barrier perception in school bullying” refers to the subjective assessment by adolescents, as non-direct participants in bullying incidents (neither bullies nor victims), of cognitive, emotional, social, and situational factors that hinder their implementation of protective intervention behaviors when witnessing school bullying. Based on Protection Motivation Theory and Ecological Systems Theory, this barrier perception encompasses two interconnected but conceptually distinct dimensions: personal risk and fear perception (threat appraisal process, focusing on personal safety threats and social costs that intervention might bring) and intervention efficacy and outcome uncertainty (coping appraisal process, focusing on the effectiveness of intervention behaviors and personal implementation capacity). This dual-dimensional structure reflects the unique psychological decision-making mechanism in bullying bystander situations, while also embodying multi-level influences from individual microsystems to socio-cultural macrosystems.

To further validate the criterion-related validity of the scale, this study selected four theoretically related variables:

Prosocial behavior: Social Cognitive Theory (Bandura, 1986) posits that situation-specific cognitions influence broader behavioral patterns. Gini et al. (2008) found that adolescents with stronger prosocial tendencies are more inclined to adopt defender roles in bullying situations.

Positive Youth Development (PYD): PYD theory suggests that healthy adolescent development depends on the interaction between internal strengths and external resources (Lerner et al., 2005). Adolescents with higher levels of positive development typically possess stronger social responsibility and intervention capabilities (Li et al., 2015).

Intentional self-regulation: In bystander intervention contexts, good self-regulation ability enables adolescents to maintain goals and flexibly adjust strategies when facing obstacles (Gestsdóttir & Lerner, 2008).

Self-esteem: Within the framework of self-efficacy theory (Bandura, 1997), individuals with higher self-esteem typically have stronger self-efficacy and can better handle challenging situations.

This study aims to develop the “Adolescent Bystander Intervention Barrier Perception Scale” (ABIBPS). Specific research questions include:

(1) What are the structural dimensions of adolescent bystander intervention barriers in school bullying?
(2) Does the developed scale demonstrate measurement invariance across different age groups of adolescents?
(3) What is the relationship between bystander intervention barrier perceptions and theoretically related variables?

## 2. Method

### 2.1. Research Procedure

This study employed a multi-stage design, including: (1) conceptual framework construction; (2) qualitative interviews and initial item development; (3) item analysis and exploratory factor analysis; (4) confirmatory factor analysis and measurement invariance testing; (5) criterion-related validity testing.

The study has been approved by the university ethics committee (Approval No. 2024031301).

### 2.2. Qualitative Interview Study

#### 2.2.1. Participants and Procedure

Using purposive sampling, 8 students were selected from each of three schools, resulting in a total sample of 24 adolescents (12 males, 12 females; age range = 10–14 years, *M* = 12.7, *SD* = 0.49) who participated in semi-structured interviews. These three schools are the same as those described in the subsequent quantitative study (Samples 1–3), namely public middle school A, public middle school B, and public middle school C, all located in provincial cities. The selection of these schools was based on several considerations: first, they represent the diversity of school environments in urban areas of China, including both urban center and suburban schools; second, they provide representative variation in terms of student family socioeconomic status; third, school administrators demonstrated a positive and cooperative attitude toward school bullying research, which ensured the smooth implementation of the study. Participants were recruited through a combination of school counselor recommendations and voluntary student registration. To ensure sample diversity, a maximum variation sampling strategy was adopted, including students who (a) had intervened in bullying, (b) wanted to intervene but did not act, (c) witnessed bullying but did not consider intervention, and (d) held unique perspectives on bullying issues.

Interviews were conducted in school counseling rooms and lasted approximately 45 to 60 min. Participants and their guardians were provided with informed consent forms detailing the study’s purpose, procedures, potential risks and benefits, confidentiality measures, and principles of voluntary participation, including the option to withdraw at any time. Interviews commenced only after informed consent was obtained.

Interviews were conducted by two psychology doctoral students trained in qualitative research methods, both with backgrounds in adolescent psychological counseling and bullying intervention research. All interviewers received specialized training in semi-structured interview techniques and sensitive topic discussion with adolescents before formal interviews. Interviews centered around core areas: open exploration of bullying intervention barriers (e.g., “Please recall, when you saw a classmate being bullied, did you consider helping him/her? What made you hesitate or worry?”), exploration based on Protection Motivation Theory (e.g., “What risks do you think you would face if you helped a bullied classmate? How significant are these risks?”), and in-depth discussion of specific barrier situations (e.g., “Please provide examples of specific difficulties or challenges you believe would be faced when implementing bullying protection behaviors. Can you share a personal experience or observation?”).

#### 2.2.2. Interview Data Analysis and Results

Thematic analysis was independently conducted by two psychology doctoral students trained in qualitative research methods. The analysis was facilitated using NVIVO 12 qualitative analysis software. We adopted Braun and Clarke’s (2006) six-step thematic analysis framework: (1) familiarizing with data: repeatedly reading interview records, making preliminary notes; (2) generating initial codes: systematically generating initial codes for the entire dataset; (3) searching for themes: integrating codes into potential themes; (4) reviewing themes: checking the correspondence between themes and coded excerpts, generating thematic maps; (5) defining and naming themes: refining and clearly defining each theme; (6) producing the report: selecting typical quotations, connecting analysis with research questions and literature. The two coders first independently coded interview records, reaching preliminary agreement before continuing to code the remaining interviews. Regular meetings were held during the coding process to discuss discrepancies until consensus was reached, indicating good reliability of the coding.

Analysis identified six main themes: personal safety concerns, social evaluation worries, intervention efficacy doubts, situational uncertainty, lack of support systems, and culture-specific barriers. These six themes can be categorized into two core barriers: one related to personal risk and emotional reactions (themes one, two, and part of six), and the other related to intervention efficacy and outcome uncertainty (themes three, four, five, and part of six). This aligns highly with the dual appraisal processes in Protection Motivation Theory—threat appraisal and coping appraisal—providing qualitative support for the scale’s two-dimensional structure.

### 2.3. Initial Scale Development

Based on the literature review in the Introduction (Section 1) and interview results, we adopted a systematic item development approach, including five steps:

(1) Theme-to-theory mapping: We systematically mapped the six themes identified in interviews to core constructs in Protection Motivation Theory and Ecological Systems Theory. For example, “personal safety concerns” and “social evaluation worries” themes corresponded to the “threat appraisal” process in Protection Motivation Theory; “intervention efficacy doubts” and “situational uncertainty” themes corresponded to the “coping appraisal” process; “lack of support systems” and “culture-specific barriers” were explained through different levels of Ecological Systems Theory (microsystem, mesosystem, exosystem, and macrosystem).
(2) Item development: Based on the theoretical importance of each dimension and frequency mentioned in interviews, we preliminarily determined two core dimensions: “Personal Risk and Fear Perception” and “Intervention Efficacy and Outcome Uncertainty.” For each dimension, we planned to develop 12–15 items to ensure content coverage and space for subsequent selection. Item distribution covered all sub-themes.
(3) Item writing: Specific items were written based on original interview expressions. For example, one student’s expression “I’m afraid that standing up to protect might make the bully more crazy” was transformed into the scale item “I worry that helping a bullied classmate might make the bully angrier and do worse things.”; another student’s mention of “I worry there’s no appropriate timing to protect others” was transformed into the scale item “I don’t know when is the best time to help.”
(4) Expert review and revision: Three psychology professors and three psychology doctoral students participated in item evaluation as independent expert reviewers, none of whom were authors of this study, ensuring objectivity of the assessment. Evaluation content included item content representativeness, clarity of expression, age appropriateness, and theoretical relevance.
(5) Cognitive interviews and language optimization: Participants in cognitive interviews were 10 students in the target age range (10–14 years), including 5 boys and 5 girls, all from schools different from the formal sample. These students had diversity in academic performance, family background, and socioeconomic status, ensuring item applicability to students from different backgrounds. Cognitive interviews employed the “think-aloud” technique, requiring students to verbally express their thinking process when answering each item, to assess item comprehensibility and the answering process.

Through these steps, 28 initial items were ultimately formed.

### 2.4. Participants

The present study employed a combination of convenience sampling and cooperative school selection to recruit participants. Specifically, the research team selected cooperating schools based on four criteria: (1) representativeness of school types, covering both urban and suburban schools to reflect the diversity of Chinese urban school environments; (2) heterogeneity of students’ family socioeconomic status (SES) to ensure the sample represented adolescents from diverse backgrounds; (3) administrative support for school bullying research to facilitate smooth data collection; and (4) geographical accessibility for the research team to conduct field surveys. Ultimately, three public schools located in a provincial capital city in Northern China were selected, comprising two urban middle schools and one suburban school.

Data were collected via paper-and-pencil questionnaires, with classes within each school randomly selected for testing. Exclusion criteria for data cleaning included: (1) incomplete questionnaires; and (2) obvious response patterns (e.g., selecting the same option for all items). Based on these procedures, three distinct samples were established for different analytical purposes.

Sample 1 was recruited from School A, a medium-sized public middle school located in the urban district, serving primarily urban students. Out of 400 distributed questionnaires, 388 valid responses were obtained (response rate = 97.0%). This sample consisted of 388 middle school students (195 males, 193 females; age range = 12–14 years, *M* = 12.8, *SD* = 0.47). Data from Sample 1 were utilized for item analysis and exploratory factor analysis (EFA).

Sample 2 was drawn from School B, a large public middle school also situated in the urban district. This institution features a relatively diverse student background, including families from various socioeconomic statuses. Out of 500 distributed questionnaires, 474 valid responses were obtained (response rate = 94.8%). This sample comprised 474 middle school students (241 males, 233 females; age range = 11–14 years, *M* = 12.8, *SD* = 0.62). Data from Sample 2 were used for confirmatory factor analysis (CFA) and measurement invariance analysis.

Sample 3 was collected from School C, a suburban public school where students are mainly drawn from the local community. Out of 600 distributed questionnaires, 547 valid responses were obtained (response rate = 91.2%). This sample included 547 upper elementary school students (276 males, 271 females; age range = 10–12 years, *M* = 11.6, *SD* = 0.35). Similar to Sample 2, data from this sample were employed for confirmatory factor analysis (CFA) and measurement invariance analysis.

The use of three distinct groups serves several methodological purposes. Employing separate samples for exploratory and confirmatory factor analyses helps to minimize sample-specific bias and enhances the robustness of the findings. Including both upper elementary and middle school students allows for examination of the scale’s applicability and measurement invariance across different developmental stages. Additionally, selecting samples from schools with varying demographic and socioeconomic backgrounds strengthens the external validity of the study and supports the generalizability of the scale across diverse educational environments.

### 2.5. Measures

#### 2.5.1. Adolescent Bystander Intervention Barrier Perception Scale (Self-Developed)

Based on a comprehensive literature review and interview findings, an initial pool of 28 items was generated. A 7-point Likert scale (1 = *strongly disagree*, 7 = *strongly agree*) was employed to capture participants’ responses. This 7-point format was deliberately chosen over shorter scales (e.g., 5-point) to maximize psychometric sensitivity. Methodological research indicates that 7-point scales offer greater response differentiation, allowing for a more precise expression of attitude intensity and resulting in data distributions that more closely approximate normality, which facilitates robust parametric statistical analysis (Bollen, 1989; Preston & Colman, 2000). Furthermore, this format has been shown to demonstrate superior reliability and discriminating power while maintaining high respondent acceptance (Preston & Colman, 2000). Crucially, for the study’s adolescent population (aged 10–14), a 7-point scale strikes an optimal balance by providing sufficient nuance without imposing the excessive cognitive burden associated with larger response options (Mellor & Moore, 2014). Higher scores represent higher levels of perceived intervention barriers. Following item analysis and exploratory factor analysis, the scale was refined to a final 10-item instrument comprising two factors.

#### 2.5.2. Criterion-Related Variable Measurement Tools

(1) Prosocial Behavior Scale. We used the prosocial behavior subscale from the student self-report version of the Strengths and Difficulties Questionnaire, revised by Du et al. (2006), consisting of 5 items, with a 3-point scale from “not true” to “certainly true.”
(2) Positive Youth Development Scale (PYD-VSF). We used the very short form of the Positive Youth Development Scale Chinese version, revised by Huang et al. (2022), covering competence, confidence, character, caring, and connection. Dimension scores are the average of the items in each dimension, and the PYD total score is the average of all items, with higher scores indicating higher overall PYD levels. Items are rated on a 5-point Likert scale.
(3) Intentional Self-Regulation Scale. We used the Intentional Self-Regulation Questionnaire developed by Gestsdóttir and Lerner (2007) and revised by W. Z. Dai et al. (2010). This questionnaire contains three dimensions: goal selection, goal optimization, and goal compensation, with a total of 9 items. It uses a 5-point scale (1 = “not at all like me,” 5 = “very much like me”), with higher total scores indicating better intentional self-regulation ability.
(4) Self-Esteem Scale. Self-esteem was measured using the Chinese version of the Rosenberg Self-Esteem Scale (Rosenberg, 1965), with the Chinese version from the Commonly Used Psychological Assessment Manual (3rd edition) provided by X. Y. Dai et al. (2023). It consists of 10 items with a 4-point rating scale, with higher total scores indicating higher self-esteem levels.

### 2.6. Data Analysis

SPSS 27.0, Mplus 8.3, and SmartPLS 4.1 software were used for data analysis, including: item analysis, exploratory factor analysis, confirmatory factor analysis, measurement invariance testing, and criterion-related validity analysis.

## 3. Results

### 3.1. Item Analysis

Through high-low group difference tests and item-total correlation analysis, all items demonstrated good discriminability and significant positive correlations with the total score.

### 3.2. Exploratory Factor Analysis

We conducted an exploratory factor analysis (EFA) on all 28 initial items using Principal Axis Factoring (PAF) with Promax rotation. PAF was selected because it is better suited to uncover the latent structure underlying observed variables (Fabrigar et al., 1999), and Promax rotation permits correlated factors, consistent with our theoretical expectation of interrelated constructs. The number of factors was determined using the Kaiser criterion (eigenvalues > 1), parallel analysis, and inspection of the scree plot.

The initial analysis indicated a two-factor solution explaining 67% of the total variance, and the factor structure was theoretically interpretable. However, several items exhibited psychometric issues, including low communalities, low primary loadings, or substantial cross-loadings.

Based on the following a priori criteria, we identified and removed suboptimal items in a single pass:

(1) Communality < 0.50; (2) All factor loadings < 0.60; (3) Salient cross-loadings on multiple factors with a cross-loading difference < 0.30; (4) In addition, given the adolescent sample and our emphasis on brevity and usability, we constrained the number of items to no more than 7 per factor and targeted a total length of 10 items.

Applying these criteria, we retained 10 items from the original pool of 28, balancing practical brevity with adequate content coverage. We then conducted a second EFA on the remaining 10 items. The results supported a clearer two-factor structure, with cumulative variance explained increasing to 76.30%. The final solution comprised 4 items for the first factor and 6 items for the second factor. Table 1 presents the factor loadings of the retained items. Loadings on their respective factors ranged from 0.76 to 0.85, indicating strong item–factor associations.

The first dimension was named “Personal Risk and Fear Perception,” primarily focusing on bystanders’ perception of direct risks and emotional reactions they might personally experience; the second dimension was named “Intervention Efficacy and Outcome Uncertainty,” primarily focusing on bystanders’ perception of the effectiveness of intervention behaviors themselves and the uncertainty of consequences.

These two dimensions align highly with the dual appraisal processes in Protection Motivation Theory (Rogers, 1975): “Personal Risk and Fear Perception” corresponds to the “threat appraisal” process, reflecting assessment of potential negative consequences of intervention; “Intervention Efficacy and Outcome Uncertainty” corresponds to the “coping appraisal” process, reflecting judgment of intervention effectiveness and one’s own abilities.

### 3.3. Confirmatory Factor Analysis

Results showed that the two-factor model fitted well in both samples. See Table 2.

#### 3.3.1. Structural Validity

In Samples 2 and 3, the composite reliability (CR) and average variance extracted (AVE) for both dimensions reached satisfactory levels. See Table 3.

#### 3.3.2. Criterion-Related Validity

Partial Least Squares Structural Equation Modeling (PLS-SEM) was used for data analysis. The choice of PLS-SEM over covariance-based structural equation modeling (CB-SEM) was based on the following considerations:

The choice of PLS-SEM aligns with the research goals of the present study. Our primary focus was to predict and explain relationships between variables rather than to conduct strict theory testing. PLS-SEM is particularly suited for studies emphasizing model prediction performance and explanatory power, whereas CB-SEM is typically used for theory testing and assessing overall model fit (Rigdon, 2012).

In SmartPLS, the default algorithm was used for model estimation, with maximum iterations set to 300 and a stop criterion of 0.0000001. The significance and confidence intervals of path coefficients were calculated through Bootstrap resampling (5000 resamples). Significance was determined through Bootstrap confidence intervals; if the 95% confidence interval did not include 0, the effect was considered significant (Mao et al., 2025).

Criterion-related validity analysis results showed that the Adolescent Bystander Intervention Barrier Perception Scale and its two dimensions were significantly negatively correlated with all criterion variables (*p* < 0.001). See Table 4.

All of these correlation coefficients were statistically significant. According to Cohen’s (1988) effect size standards, values of r = 0.10, r = 0.30, and r = 0.50 correspond to small, medium, and large effects, respectively. In this study, the correlation between ABIBP and self-esteem (r = −0.33) approaches a medium effect size. Correlations with positive youth development (r = −0.23) and prosocial behavior (r = −0.24), though smaller, are still meaningful within the context of educational and developmental psychology research. Notably, the correlation with intentional self-regulation was the smallest (r = −0.19); although statistically significant, this represents a small effect size and may suggest that the relationship between self-regulation ability and barrier perception is influenced by other moderating factors. In the field of bullying intervention research, even relatively small correlation coefficients may have cumulative practical significance.

### 3.4. Age Measurement Invariance of the Adolescent Bystander Intervention Barrier Perception Scale

Mplus 8.3 was used for invariance testing, with Samples 2 and 3 for cross-age measurement invariance analysis. See Table 5.

The ΔCFI of each level of invariance model indicates that the scale has good measurement invariance in upper elementary and middle school student groups, including configural invariance, metric invariance, scalar invariance, and strict invariance (Chen, 2007; Cheung & Rensvold, 2002). This indicates that the scale has the same psychological meaning across different age stages, allowing direct comparison of scores between adolescents at different age stages.

### 3.5. Gender Measurement Invariance of the Adolescent Bystander Intervention Barrier Perception Scale

Considering the importance of gender differences in school bullying research, we tested the measurement invariance of the scale across different gender groups. The procedure was identical to the previous section, with results shown in Table 6.

According to the standards proposed by Cheung and Rensvold (2002) and Chen (2007), when ΔCFI ≤ 0.01, it can be considered that there is no significant difference between nested models, supporting the establishment of measurement invariance. As seen from Table 5, the absolute values of ΔCFI for all levels of invariance models are less than 0.01, indicating that the scale has good measurement invariance across different gender groups. This suggests that the scale has the same psychological meaning for different genders, allowing direct comparison of scores between adolescents of different genders.

This result provides a reliable measurement foundation for future research on gender differences in bullying intervention. The structural and measurement characteristics of the scale remain stable across different gender groups, ensuring the comparability of research results and facilitating subsequent exploration of potential substantive differences in intervention barrier perceptions between male and female adolescents.

## 4. Discussion

### 4.1. Dimensional Structure and Theoretical Significance of Adolescent Bystander Intervention Barriers in School Bullying

Through exploratory and confirmatory factor analyses, this study confirmed a two-factor structure for adolescent bystander intervention barrier perceptions in school bullying: “Personal Risk and Fear Perception” and “Intervention Efficacy and Outcome Uncertainty.” This structure is not only supported by statistical data but also has a profound theoretical foundation, highly consistent with our expectations based on multiple theoretical frameworks.

First, this two-factor structure clearly corresponds to the dual appraisal processes in Protection Motivation Theory (Rogers, 1975)—threat appraisal and coping appraisal. The “Personal Risk and Fear Perception” dimension reflects adolescents’ threat appraisal of potential negative consequences of intervention (such as retaliation, social exclusion, etc.); the “Intervention Efficacy and Outcome Uncertainty” dimension reflects coping appraisal of intervention effectiveness and personal capabilities. DeSmet et al. (2016), in applying Protection Motivation Theory to study cyberbullying bystander behavior, similarly found a dual appraisal structure, supporting the applicability of this theoretical framework in bullying intervention contexts.

Specifically, according to Protection Motivation Theory, when individuals face threatening situations (such as witnessing bullying), they engage in two parallel appraisals: first, threat appraisal, including assessment of threat severity, personal vulnerability, and internal and external rewards for inaction; second, coping appraisal, including assessment of response efficacy, self-efficacy, and response costs. In this study, the “Personal Risk and Fear Perception” dimension primarily corresponds to “vulnerability” (possibility of becoming the next victim) and negative aspects of “internal and external rewards” (such as losing social status) in threat appraisal; the “Intervention Efficacy and Outcome Uncertainty” dimension primarily corresponds to “response efficacy” (whether intervention can be effective) and “self-efficacy” (whether one has the capacity to intervene) in coping appraisal. This correspondence indicates that the scale’s two-dimensional structure is not merely a statistical classification but a theoretical reflection of adolescents’ intervention decision-making psychological processes.

Second, this structure reflects the multi-level influences emphasized in Ecological Systems Theory (Bronfenbrenner, 1979). The “Personal Risk and Fear Perception” dimension focuses more on internal individual responses and direct interaction threats in the microsystem; the “Intervention Efficacy and Outcome Uncertainty” dimension involves assessment of broader environmental factors, including external support systems and situational complexity. Research applying an ecological systems perspective to bullying intervention similarly emphasizes the distinction between individual internal factors and environmental factors, resonating with our findings.

From an Ecological Systems Theory perspective, adolescent intervention behaviors are influenced by multi-level environmental factors from microsystems (such as individual internal traits, direct interpersonal interactions) to macrosystems (such as cultural values). The two-dimensional structure in our study reflects this multi-level influence: “Personal Risk and Fear Perception” primarily involves microsystem-level barriers (individual internal fears and direct interpersonal threats), while “Intervention Efficacy and Outcome Uncertainty” more involves interactions between microsystems and mesosystems (such as insufficient school support environments, lack of intervention skills). This distinction helps us more comprehensively understand the ecological nature of intervention barriers, recognizing that intervention behaviors are influenced not only by individual factors but also shaped by broader social environments.

In applying Ecological Systems Theory to analyze bystander intervention barriers, it is necessary to further clarify the role and position of the exosystem in the present study. According to Bronfenbrenner (1979, 1994), the exosystem encompasses social settings in which the individual does not directly participate but which nonetheless exert an indirect influence on their development, such as school policy systems, community service structures, and parental workplaces. In the context of school bullying, exosystem-level factors involving the presence, content, and implementation fidelity of anti-bullying policies, along with the quality of teacher training systems, may indirectly affect bystanders’ intervention decisions by influencing school climate and normative beliefs (Hong & Espelage, 2012; Hall, 2017).

The present study was designed to primarily focus on microsystem-level factors (internal psychological responses and threat perceptions in direct interpersonal interactions) and mesosystem-level factors (immediate social interactions of adolescents within school contexts). This focus was based on several methodological and theoretical considerations. First, the core objective of this study was to develop a psychometric instrument measuring individuals’ subjective perceptions of barriers, rather than to evaluate the influence of external policies or institutional environments. Barrier perception, as an individual-level psychological construct, primarily reflects adolescents’ subjective assessment of potential risks and efficacy associated with intervention behaviors, which corresponds more directly to the operational mechanisms of microsystems and mesosystems. Second, the threat appraisal and coping appraisal processes emphasized in Protection Motivation Theory are essentially internal cognitive-affective processes at the individual level, making direct measurement at this level more appropriate. Third, from a measurement perspective, adolescents’ perceptions of exosystem factors such as school policies may be subject to information asymmetry—research has shown that students often lack accurate knowledge about whether anti-bullying policies exist in their schools (Hall, 2017), which could compromise measurement validity.

Nevertheless, we fully recognize the important role that exosystem factors play in shaping bystander intervention behavior. Research indicates that school-level anti-bullying policies can influence student behavior through multiple pathways, including explicitly prohibiting certain behaviors (such as threatening, harassing others, or retaliating against students who report incidents), requiring teachers to report bullying incidents, establishing investigation procedures, and specifying consequences for perpetrators (Hall, 2017). These policy factors serve as upstream interventions that provide an institutional foundation for downstream individual-level interventions (Hall, 2017). Concurrently, school climate, as a mediating variable between exosystem and microsystem, has been demonstrated to be significantly associated with bullying incidence rates and bystander behavior patterns (Bradshaw et al., 2021).

Based on these considerations, the absence of direct exosystem variables indeed constitutes a limitation of the present study, which we have explicitly acknowledged in Section 4.5 “Research Limitations and Future Directions.”

This two-dimensional structure possesses not only theoretical significance but also close relevance to the actual behavioral manifestations of bystanders in school bullying contexts. Understanding how high versus low scores on each dimension manifest in actual bullying episodes is crucial for translating theory into practice.

For the “Personal Risk and Fear Perception” dimension, individuals with high scores typically exhibit the following characteristics when witnessing bullying incidents: they tend to physically distance themselves from the conflict scene and avoid eye contact with perpetrators; at the social level, they display pronounced “withdrawal behaviors,” (Thornberg et al., 2012), such as pretending not to see the incident, looking down at their phones, or quickly leaving the scene. These adolescents may experience intense internal emotional responses (such as anxiety and fear), but their overt behavior presents as passive bystanding or the “outsider” role (Salmivalli et al., 1996). In classroom contexts, those with high risk perception may avoid discussing bullying incidents in public, fearing that they will be viewed by peers as “tattlers” and subsequently experience social exclusion (Pozzoli & Gini, 2013).

For the “Intervention Efficacy and Outcome Uncertainty” dimension, individuals with high scores display different characteristics in bullying situations: they may possess a certain willingness to intervene, but demonstrate obvious hesitation and delay when taking actual action (Jenkins & Nickerson, 2019). Specific behavioral manifestations include: repeatedly assessing the situation when bullying occurs, thereby missing optimal intervention opportunities; adopting ambiguous or indirect intervention approaches (such as calling out from a distance rather than directly intervening); and exhibiting regret and self-reproach after the incident (“I should have done something”). The core dilemma for these adolescents stems not from concerns about personal safety, but from uncertainty about “how to intervene effectively” and “whether intervention will be useful.” In classroom discussions, they may raise doubts such as “I don’t know how to help” or “I’m not sure if the teacher will do anything about it.”

The behavioral manifestations of these two dimensions have important diagnostic value. In practical school bullying prevention work, educators can identify students’ primary barrier types by observing their typical response patterns: if students display obvious avoidance and fear responses, the focus should be on “Personal Risk and Fear Perception”; if students display a gap between willingness and action, the focus should be on “Intervention Efficacy and Outcome Uncertainty.” This behavior-based differential diagnosis provides a practical foundation for developing personalized intervention strategies.

### 4.2. Psychometric Properties and Application Value of the Adolescent Bystander Intervention Barrier Perception Scale

The Adolescent Bystander Intervention Barrier Perception Scale developed in this study demonstrated excellent psychometric properties. First, reliability indicators all reached acceptable levels, indicating good internal consistency and stability of the scale.

Second, the scale’s validity was supported by multiple lines of evidence. Content validity was ensured through expert evaluation and cognitive interviews; structural validity was verified through EFA and CFA; convergent and discriminant validity were supported through correlation analyses with related constructs.

Third, measurement invariance analysis indicated that the scale has good measurement invariance across different age groups and gender groups of adolescents, including configural invariance, metric invariance, scalar invariance, and strict invariance. This result is particularly important as it indicates that the construct measured by the scale has the same psychological meaning across different groups, providing a solid foundation for cross-group comparisons. Additionally, from a developmental psychology perspective, this result has multiple implications. Primarily, it indicates that intervention barriers as a psychological construct have the same conceptual structure across different age stages; that is, the basic dimensions of intervention barrier perceptions are consistent between upper elementary school students and middle school students. This aligns with findings from Eisenberg et al.’s (2006) research on prosocial behavior development, which suggests that although age growth may affect the manifestation forms of prosocial behavior, its basic motivational and barrier structures remain relatively stable from middle childhood to adolescence.

The development of this scale fills an important gap in school bullying research. Previous research has mainly focused on factors promoting bystander intervention behaviors, such as empathy, group norms, and related promotive influences, with less systematic exploration of hindering factors (Nickerson et al., 2014; Pozzoli & Gini, 2013; Salmivalli, 2014; Thornberg & Jungert, 2013; Sjögren et al., 2024). This scale provides a reliable tool for assessing these hindering factors, not only contributing to in-depth theoretical research but also providing guidance for practical interventions.

From a theoretical research perspective, the scale can be used to explore relationships between intervention barriers and other psychological constructs (such as moral disengagement, group norms, self-esteem levels, etc.), deepening our understanding of bystander behavioral decision-making processes. From a practical application perspective, the scale can be used to assess the effects of school bullying intervention programs, especially the effects of interventions targeting bystander roles, and can also be used to screen individuals facing high levels of intervention barriers, providing them with targeted support.

### 4.3. Relationships Between Adolescent Bystander Intervention Barrier Perceptions and Related Variables and Their Theoretical Implications

The criterion-related validity analysis revealed that adolescent bystander intervention barrier perceptions were significantly and negatively correlated with multiple theoretically relevant variables, including prosocial behavior, positive youth development (PYD), intentional self-regulation, and self-esteem. These correlational patterns not only establish the scale’s concurrent validity but also offer critical insights into the positioning of intervention barriers within the broader network of adolescents’ psychosocial development.

From the perspective of social cognition and development, the negative correlations between barrier perceptions and both prosocial behavior (*r* = −0.24) and PYD indicators (*r* = −0.23) align with theoretical expectations. Consistent with Social Cognitive Theory and findings by Gini et al. (2008), these results suggest that situation-specific cognitions in bullying contexts are intrinsically linked to broader behavioral patterns. Furthermore, the findings support the core hypothesis of the PYD framework: the accumulation of internal strengths and external resources facilitates the management of developmental challenges (Lerner et al., 2005). Consequently, intervention barrier perceptions should not be viewed as isolated situational responses but rather as constructs nested within adolescents’ overall developmental trajectories, closely tied to core developmental assets such as competence and character. Notably, the “Personal Risk and Fear Perception” dimension demonstrated slightly stronger associations with these broad social development indicators, suggesting that emotion-based barriers are particularly sensitive to an individual’s general social functioning.

Regarding the self-system, the negative correlation between barrier perceptions and intentional self-regulation (*r* = −0.19), while statistically significant, was relatively weak. This supports the expectation from self-regulation theory that abilities in goal selection and optimization aid in addressing behavioral hurdles (Gestsdóttir & Lerner, 2008). However, the modest magnitude of this association implies that while self-regulation contributes to overcoming barriers, its influence may be moderated by other factors, such as situational ambiguity or peer pressure. Conversely, the robust negative correlation with self-esteem (*r* = −0.33) strongly validates self-efficacy theory, which posits that positive self-evaluation enhances confidence in addressing challenges (Bandura, 1997).

A nuanced examination of the sub-dimensions reveals distinct psychological mechanisms. The “Intervention Efficacy and Outcome Uncertainty” dimension showed a stronger correlation with self-esteem (*r* = −0.35) compared to the “Personal Risk and Fear Perception” dimension (*r* = −0.30). This differentiated pattern suggests that self-esteem mitigates intervention barriers primarily by bolstering efficacy beliefs and reducing doubts about outcomes, rather than merely dampening risk perceptions. This finding further substantiates the two-factor structure of the scale, indicating that different types of barriers operate through distinct pathways.

Integrating these findings allows for the construction of a comprehensive theoretical model: intervention barrier perceptions serve as a composite reflection of adolescents’ self-systems (e.g., self-esteem, self-regulation), social functioning (e.g., prosocial tendencies), and positive developmental resources. This integrated model expands the understanding of bystander behavior and carries significant practical implications. First, it validates barrier perception as a critical entry point for intervention. Second, it highlights the necessity of holistic approaches; anti-bullying education should be integrated with efforts to promote overall positive youth development. Finally, the differential correlation strengths suggest targeted intervention priorities—for instance, interventions enhancing self-esteem may yield more direct effects on reducing efficacy-related barriers than those focusing solely on self-regulation skills.

### 4.4. Theoretical Contributions and Practical Implications

#### 4.4.1. Theoretical Contributions

(1) By integrating Protection Motivation Theory and Ecological Systems Theory, this study offers a multi-level framework of bystander intervention barriers, moving beyond single-perspective models and capturing the complexity of bystander behavior (Salmivalli, 2014).
(2) Empirical support for a two-factor structure—Personal Risk Perception and Efficacy Uncertainty—clarifies distinct yet interactive cognitive–affective processes underpinning bystander decisions, strengthening the psychological mechanism evidence base.
(3) Mapping links between barrier perceptions and developmental indicators (e.g., positive youth development, self-regulation) extends theory connecting bystander behavior to adolescent development, aligning with calls to embed bullying intervention within a PYD framework (Espelage, 2014). This suggests moving beyond narrow skills training toward broader developmental promotion.
(4) Establishing measurement invariance enables examination of developmental trajectories in intervention barriers and supports future longitudinal work.

#### 4.4.2. Practical Implications

The study provides a culturally adapted, psychometrically sound tool for Chinese adolescents to assess baseline barriers, track change during interventions, and evaluate outcomes.

(1) Multidimensional intervention strategies:
  (i) For Personal Risk and Fear Perception: establish safety-support systems; encourage collective (vs. solo) intervention; teach safe/indirect strategies and help-seeking.
  (ii) For Efficacy and Outcome Uncertainty: offer clear stepwise guidance on when/where/how to act; present successful cases to build confidence; use role-play to strengthen skills for uncertain situations.
(2) Measurement invariance supports comparable assessment across ages and genders, enabling tailored, data-driven programming for different subgroups.
(3) Integrative development approach: pair barrier reduction with resource building (e.g., self-esteem, social skills, moral education, self-regulation), consistent with PYD principles emphasizing holistic asset development (Lerner et al., 2005).
(4) School ecological change: beyond individual-level training, modify the broader school ecology—clear anti-bullying norms, supportive peer culture, teacher intervention support, and a safe climate—consistent with social–ecological models (Swearer & Hymel, 2015).
(5) Assessment-guided intervention: use the scale for needs assessment and iterative evaluation—pretest to identify barriers, mid-course monitoring to adjust strategies, and posttest to appraise effects—aligning with evidence-based psychosocial intervention standards (Durlak et al., 2011).

### 4.5. Research Limitations and Future Directions

This study has several limitations that need to be addressed in future research. First, although we used cross-regional samples to enhance the representativeness of results, the samples were still limited to urban areas in eastern and northern China. Future research should expand sample coverage, especially including adolescents from western, southern, and rural areas, as well as conducting cross-cultural research. Second, although this study conducted test–retest reliability analysis following questionnaire development conventions, the tracking period was relatively short. Future research should conduct longer-term tracking to better understand the developmental trajectory of intervention barrier perceptions.

Although this study confirmed measurement invariance across different gender groups, future research could further explore substantive differences in intervention barrier manifestations between male and female adolescents and the formation mechanisms of these differences. Additionally, future research could also explore relationships between more detailed bystander role classification frameworks and intervention barriers, deepening understanding of unique barrier patterns faced by different types of bystanders.

Furthermore, the present study primarily focused on the microsystem and mesosystem levels within the Ecological Systems Theory framework, without fully incorporating exosystem-level variables. The exosystem includes school anti-bullying policies, teacher training systems, and community support structures—social environmental factors that indirectly influence adolescent development (Bronfenbrenner, 1994). Existing research indicates that school-level policy interventions, as systemic upstream measures, can influence overall school practices by regulating the behaviors of students, teachers, and administrators (Hall, 2017). However, the specific mechanisms through which policy factors affect adolescent bystanders’ intervention decisions require further investigation. On one hand, the presence of policies may reduce bystanders’ perceived intervention barriers by fostering a supportive school climate, clarifying behavioral norms, and providing protection for reporters; on the other hand, if policies are poorly implemented or students lack awareness of them, the potential positive effects may be attenuated (Hall, 2017). Future research should adopt multilevel research designs to systematically examine how school policy variables (such as the comprehensiveness of policy content, consistency of implementation, and protective measures for reporters) affect individual-level barrier perceptions through their influence on school climate. Such investigations would provide empirical evidence for developing more effective anti-bullying policies.

## 5. Conclusions

(1) Grounded in Protection Motivation Theory and Ecological Systems Theory, this study is the first to develop a reliable and valid instrument from the perspective of perceived barriers, thereby shifting the research lens and providing a new theoretical framework and practical tool for bullying research. The measure establishes a two-factor structure comprising “Personal Risk and Fear Perception” and “Intervention Efficacy and Outcome Uncertainty.” Specifically, “Personal Risk and Fear Perception” corresponds to the threat appraisal process in Protection Motivation Theory, reflecting bystanders’ perception of personal safety threats and social costs that intervention might bring; “Intervention Efficacy and Outcome Uncertainty” corresponds to the coping appraisal process, reflecting bystanders’ judgment of the effectiveness of intervention behaviors and personal implementation capacity.
(2) The scale demonstrates strong reliability, validity, and measurement invariance, offering an effective tool for assessing adolescents’ cognitions about bystander intervention barriers. At the practical level, the scale can be used to inform school-based bullying prevention efforts and practices that promote positive youth development. Specifically, educators can utilize this scale for needs assessment to identify the primary barrier types in student populations, thereby designing targeted intervention strategies: for students with high “Personal Risk and Fear Perception,” interventions can focus on establishing safety-support systems, encouraging collective intervention, and teaching indirect help-seeking strategies to reduce their perceived risk; for students with high “Intervention Efficacy and Outcome Uncertainty,” interventions can focus on providing clear intervention guidance, presenting successful cases, and conducting role-play training to enhance their intervention confidence. Furthermore, this scale can be used to track intervention effects and evaluate the effectiveness of anti-bullying programs, supporting evidence-based school practices.

## Figures and Tables

**Table 1:** Characteristic Factors of the 2 Subscales of the ABIBP Scale.

Items	Factor Loading		Communality
	Perceived Personal Risk and Fear	Intervention Efficacy and Outcome Uncertainty	
I worry that helping bullied classmates might make the bullies angrier and do worse things.	**0.83**	0.02	0.74
I’m afraid that helping bullied classmates might make other students unwilling to play with me.	**0.82**	0.16	0.79
I’m a bit scared and prefer not to get involved in other people’s troubles.	**0.79**	0.10	0.81
I feel that if I help, it might make the situation worse.	**0.79**	0.08	0.76
I don’t know when would be the best time to offer help.	0.07	**0.85**	0.74
I worry that if I help, those bullies might bully others even more.	0.02	**0.83**	0.75
If no one supports me, I would feel lonely and unsure of what to do.	0.05	**0.79**	0.78
If I’m uncertain about what happened, I might not know whether I should help or not.	0.11	**0.79**	0.7
I worry that my help might be useless, or others might think I’m meddling in their business.	0.10	**0.78**	0.78
If the bullied classmate doesn’t want my help, I would feel confused and sad, not knowing what to do.	0.11	**0.76**	0.79
Internal Consistency Reliability	0.90	0.94	

Note. Primary factor loadings are displayed in bold. Cross-loadings are displayed to demonstrate the cleanness of the factor structure. All cross-loadings were below the 0.30 threshold, indicating minimal overlap between factors.

**Table 2:** Fit Indices of the Two-Factor Structure of the ABIBP Scale.

	*x* ^2^	*df*	RMSEA	CFI	TLI	SRMR
Middle School (Sample 2)	76.041	34	0.051	0.976	0.969	0.027
Upper Elementary (Sample 3)	110.296	34	0.064	0.965	0.954	0.027

**Table 3:** Results of CFA and construct validity.

Items	Perceived Personal Risk and Fear		Intervention Efficacy and Outcome Uncertainty	
I worry that helping bullied classmates might make the bullies angrier and do worse things.	**0.75**	**0.81**		
I’m afraid that helping bullied classmates might make other students unwilling to play with me.	**0.84**	**0.83**		
I’m a bit scared and prefer not to get involved in other people’s troubles.	**0.89**	**0.86**		
I feel that if I help, it might make the situation worse.	**0.88**	**0.89**		
I don’t know when would be the best time to offer help.			**0.79**	**0.87**
I worry that if I help, those bullies might bully others even more.			**0.80**	**0.85**
If no one supports me, I would feel lonely and unsure of what to do.			**0.85**	**0.78**
If I’m uncertain about what happened, I might not know whether I should help or not.			**0.87**	**0.76**
I worry that my help might be useless, or others might think I’m meddling in their business.			**0.87**	**0.86**
If the bullied classmate doesn’t want my help, I would feel confused and sad, not knowing what to do.			**0.84**	**0.72**
CR	0.91	0.91	0.93	0.92
AVE	0.71	0.72	0.70	0.65

Note. Standardized factor loadings are displayed in bold. The first column shows the standardized loadings for Sample 2, while the second column displays the standardized loadings for Sample 3.

**Table 4:** Criterion-Related Validity of ABIBPS (*r*).

Criterion	Prosocial Behavior	PYD	Intentional Self-Regulation	Self-Esteem
ABIBP	−0.24 ***	−0.23 ***	−0.19 ***	−0.33 ***
Personal Risk and Fear Perception	−0.25 ***	−0.24 ***	−0.19 ***	−0.30 ***
Intervention Efficacy and Outcome Uncertainty	−0.23 ***	−0.22 ***	−0.19 ***	−0.35 ***

Note: *** *p* < 0.001.

**Table 5:** Measurement Invariance Testing of the ABIBP Scale.

	Model	*x* ^2^	*df*	c	RMSEA	CFI	TLI	SRMR	|ΔCFI|
Measurement invariance	configural	185.99	68	1.94	0.058	0.970	0.961	0.027	
	metric	213.35	76	1.84	0.060	0.965	0.959	0.038	0.005
	scalar	235.51	84	1.76	0.059	0.962	0.959	0.040	0.003
	residual	268.97	94	1.84	0.060	0.956	0.958	0.040	0.006
Structural invariance	variance	275.68	96	1.81	0.06	0.955	0.958	0.043	0.001
	covariance	303.67	97	1.82	0.065	0.948	0.952	0.047	0.007
	latent	334.16	99	1.80	0.068	0.941	0.946	0.086	0.007

Note: c is the correction factor for the MLR method, |ΔCFI| is the absolute value of ΔCFI.

**Table 6:** Gender Measurement Invariance Testing of the ABIBP Scale.

	Model	*x* ^2^	*df*	c	RMSEA	CFI	TLI	SRMR	|ΔCFI|
Measurement invariance	configural	185.338	68	1.749	0.058	0.970	0.961	0.025	
	metric	198.963	76	1.660	0.056	0.969	0.963	0.027	0.001
	scalar	213.985	84	1.600	0.055	0.967	0.965	0.030	0.002
	residual	214.745	94	1.665	0.050	0.970	0.971	0.031	0.003
Structural invariance	variance	219.128	96	1.643	0.050	0.969	0.971	0.033	0.001
	covariance	218.026	97	1.651	0.049	0.969	0.972	0.033	0.000
	latent	240.395	99	1.639	0.053	0.964	0.968	0.073	0.005

## Data Availability

The data presented in this study are available on request from the corresponding author.
